# Real-World Safety, Effectiveness, and Patient-Reported Outcomes in Patients with Chronic Hepatitis C Virus Infection Treated with Glecaprevir/Pibrentasvir: Updated Data from the German Hepatitis C-Registry (DHC-R)

**DOI:** 10.3390/v14071541

**Published:** 2022-07-14

**Authors:** Markus Cornberg, Albrecht Stoehr, Uwe Naumann, Gerlinde Teuber, Hartwig Klinker, Thomas Lutz, Hjördis Möller, Dennis Hidde, Kristina Lohmann, Karl-Georg Simon

**Affiliations:** 1 Klinik für Gastroenterologie, Hepatologie und Endokrinologie, Hannover Medizinische Hochschule, 30625 Hannover, Germany; 2 IFI Medizin GmbH, 20099 Hamburg, Germany; stoehr@ifi-medizin.de; 3 UBN-Praxis, 14059 Berlin, Germany; naumann@ubn-praxis.de; 4 Practice PD Dr. med. G. Teuber, 60594 Frankfurt am Main, Germany; tcr-ffm@t-online.de; 5 Department of Internal Medicine, University Hospital Würzburg, 97080 Würzburg, Germany; klinker_h@ukw.de; 6 Infektiologikum, 60596 Frankfurt, Germany; lutz@infektiologikum.de; 7 Leberzentrum am Checkpoint, 10961 Berlin, Germany; hjoerdism@hotmail.com; 8 AbbVie Germany GmbH & Co., KG, 65189 Wiesbaden, Germany; dennis.hidde@abbvie.com (D.H.); kristina.lohmann@abbvie.com (K.L.); 9 MVZ Dres Eisenbach/Simon/ Schwarz/GbR, 51375 Leverkusen, Germany; k.-g.simon@t-online.de

**Keywords:** direct-acting antiviral, glecaprevir/pibrentasvir, hepatitis C virus, real world evidence, German Hepatitis C-Registry

## Abstract

Using data from the German Hepatitis C-Registry (Deutsche Hepatitis C-Register, DHC-R), we report the real-world safety and effectiveness of glecaprevir/pibrentasvir (GLE/PIB) treatment and its impact on patient-reported outcomes (PROs) in underserved populations who are not typically included in clinical trials, yet who will be crucial for achieving hepatitis C virus (HCV) elimination. The DHC-R is an ongoing, non-interventional, multicenter, prospective, observational cohort study on patients treated for chronic HCV infection in Germany. The data cutoff was 17 January 2021. The primary effectiveness endpoint was sustained virologic response at post-treatment Week 12 (SVR12). Safety outcomes were assessed in all patients receiving GLE/PIB. PROs were assessed using the SF-36 survey. Of 2354 patients, 1964 had valid SVR12 data (intention-to-treat analysis). Of these, 1905 (97.0%) achieved SVR12 with rates similar across the comorbidities analyzed, except for people who actively use drugs (PWUD (active)) (86.4%). Excluding those who discontinued treatment and did not achieve SVR12, or were reinfected with HCV, the rate was 99.3%, with similar results regardless of comorbidity. PWUD (active) and those with psychiatric disorders had the most meaningful improvements in PROs. Adverse events (AEs) occurred in 631/2354 patients (26.8%), and serious AEs in 44 patients (1.9%). GLE/PIB was highly effective and well tolerated in this real-world study of patient groups key to HCV elimination.

## 1. Introduction

In 2019, an estimated 58 million people worldwide were infected with chronic hepatitis C virus (HCV) [1]. In Germany, there were more than 200,000 HCV infections estimated in 2016, with approximately 29% of these lacking a formal diagnosis [2]. The World Health Organization (WHO) has set targets aiming to eliminate HCV as a public health threat by 2030, including diagnosing 90% of HCV cases, and treating 80% of eligible patients [3]. 

It is recommended that adult patients with chronic HCV infection are treated with pangenotypic direct-acting antivirals (DAAs) [4], which provide high cure rates, minimal safety concerns, and treatment durations as short as 8 weeks [5,6]. Although the emergence of these therapies has resulted in the WHO’s targets for HCV elimination becoming a realistic goal for many countries, including Germany, most are not predicted to eliminate HCV before 2050 [7].

In spite of the available treatments and progress towards HCV elimination targets, barriers still exist, particularly engaging and retaining in treatment marginalized or underserved populations who are seldom represented in clinical trials. This includes migrant populations and patients with key comorbidities such as those receiving opioid substitution therapy (OST), those with active drug use, psychiatric disorders, alcohol abuse or dependence, or human immunodeficiency virus (HIV) coinfection. Although cure is the primary goal of HCV treatment, many patients, particularly those typically classed as ‘underserved’, are looking for benefits beyond clinical cure [8]. Insights into the patient-reported outcomes (PROs) associated with HCV treatment may help efforts to improve the linkage to care among these groups of patients.

Glecaprevir/pibrentasvir (GLE/PIB) is a fixed dose, once-daily, all-oral combination DAA therapy approved by the European Medicines Agency to treat chronic HCV infection (genotypes (GT) 1–6) over 8 weeks in treatment-naïve (TN) patients without cirrhosis (NC), or with compensated cirrhosis (CC) and GT1, 2, 4–6 in pegylated-interferon- (peg-IFN) and/or sofosbuvir-experienced NC patients; 12 weeks for GT1, 2, 4–6 in peg-IFN- and/or sofosbuvir-experienced patients with CC; and 16 weeks for GT3 in peg-IFN- and/or sofosbuvir-experienced patients regardless of cirrhosis [9]. The safety and efficacy of GLE/PIB has been well established in clinical trials [10,11,12,13,14,15]; however, few real-world studies have investigated the treatment’s impact on PROs, particularly in underserved populations who are not typically included in clinical trials, yet who will be crucial for achieving HCV elimination. In these populations, such as people who inject drugs, there is a lack of linkage to care; in some cases, less than 10% of people who inject drugs who are evaluated for HCV care start antiviral treatment [16,17,18]. The use of the 8-week treatment duration of GLE/PIB may be key to managing some of these hard-to-reach patients, including those who have been incarcerated, patients with psychiatric disorders, and people who inject drugs [19].

The German Hepatitis C-Registry (Deutsche Hepatitis C-Register, DHC-R) was founded to collect data on the real-world effectiveness and safety of all available HCV treatment regimens and assess patient care [20]. The aim of the present analysis was to use data from the DHC-R to evaluate the real-world safety and effectiveness of GLE/PIB treatment and its impact on PROs in patient subgroups who are under-represented in clinical trials.

## 2. Materials and Methods

### 2.1. Study Design and Patient Population

The DHC-R is an ongoing, non-interventional, multicenter, prospective, observational cohort study on the treatment of adults with chronic HCV infection throughout Germany (Federal Institute for Drugs and Medical Devices registration number 2493; German Clinical Trials Register ID DRKS00009717) and registry enrollment began in January 2014. The data were collected from 28 May 2017 (only baseline data were collected until the approval of GLE/PIB in July 2017) until 17 January 2021 from 151 sites. The design of the registry and the inclusion/exclusion criteria have been described elsewhere [21]. The study was conducted in accordance with the Declaration of Helsinki and Good Clinical Practice guidelines and approved by the Institutional Review Board (Ethics Committee of Ärztekammer Westfalen-Lippe; reference number 2014-395-f-S).

All patients had to provide written informed consent before enrollment in the registry; enrollment took place upon screening or initiation of antiviral therapy. The decision to treat, choice of treatment, and number and frequency of study visits were at the discretion of the treating physician. The data were recorded via an electronic case report form, with plausibility checks and random onsite visits to ensure high data quality. The patients were chronically infected with HCV and treated with GLE/PIB either according to the EMA-approved label (on-label), or not (off-label). On-label and off-label treatments were analyzed separately, focusing on on-label therapy to maximize the clinical relevance and comparability of the provided safety, effectiveness, and quality of life outcome data. 

The on-label duration of GLE/PIB therapy for TN patients with CC changed for the GTs 1, 2, 4, 5, and 6 in July 2019 and GT3 in March 2020, from a duration of 12 weeks to 8 weeks. Therefore, both 8- and 12-week data in TN/CC patients treated according to the label in effect during their treatment were analyzed separately.

### 2.2. Outcomes and Endpoints

Patient demographics and clinical characteristics were recorded at baseline and included gender, age, duration of infection, SF-36 (36-Item Short Form Health Survey) mental and physical component scores, HCV genotype, treatment history, fibrosis stage, and cirrhosis status. The fibrosis METAVIR stage was determined by histologic scoring, transient elastography, and/or acoustic radiation force impulse imaging. The presence of cirrhosis was confirmed by liver biopsy (METAVIR fibrosis stage F4), transient elastography (FibroScan^®^, Echosens, France) > 12.5 kPa, ultrasound, and/or clinical evidence of cirrhosis (e.g., the presence of ascites or esophageal varices). The presence of key comorbidities (OST, active drug use, psychiatric disorders, alcohol abuse/dependence, HIV coinfection) and migrant status were also recorded and used to define populations of special interest for effectiveness analyses. 

The primary effectiveness endpoint was the percentage of patients with sustained virologic response at post-treatment Week 12 (SVR12; defined as HCV RNA ≤ 25 IU/mL 70–153 days after end of treatment (EOT)). Additional analyses included SVR12 rate in the off-label population, TN patients, and by population of special interest, and PROs at post-treatment Week 12 (PTW12). PROs were assessed using the self-administered SF-36 survey, which contains 36 questions addressing eight components of mental and physical health; the individual component scores were pooled to provide component summary scores for mental and physical health [22,23,24]. SF-36 scoring is norm-based, with scores below 50 indicating below-average health, pain, distress, or functioning [22]. A clinically relevant improvement in SF-36 score was defined as ≥2.5-point increase in mental (MCS) or physical component summary (PCS) score from baseline to PTW12.

Safety outcomes (as recorded using the Medical Dictionary for Regulatory Activities version 19) included treatment-emergent adverse events (AEs) reported per person, and clinical laboratory abnormalities (bilirubin, alanine, and aspartate aminotransferase).

### 2.3. Statistical Analysis

The data cutoff for this analysis was 17 January 2021. The total population included all patients with chronic HCV infection who received ≥ 1 dose of GLE/PIB. Baseline demographics, clinical characteristics, and safety were reported in the total population. The virologic response was assessed for all adult patients with chronic HCV infection who received ≥1 dose of GLE/PIB, completed a screening visit on or after 28 May 2017 through to 30 December 2019, and had documented virologic load data at PTW12 (effectiveness population; EP) using an intention-to-treat (EP-ITT) analysis, which excluded patients becoming lost to follow-up at the follow-up visit scheduled at PTW12 or 24 who therefore did not have SVR12 data. A modified EP-ITT (EP-mITT) analysis was also conducted that excluded patients who discontinued treatment and did not achieve SVR12, or were reinfected with HCV. The data for on- and off-label patients were analyzed separately.

Summary statistics (*n*, mean, median, standard deviation (SD), minimum, maximum) were generated for the continuous variables, and the number and percentage of patients were reported for the categorical variables. Statistical analyses comparing the baseline SF-36 component summary scores between populations of special interest were conducted using the SAS^®^ software package (version 9.4; SAS Institute Inc., Cary, NC, USA). 

## 3. Results

### 3.1. Patient Characteristics

During this period, 2354 patients were treated on-label with GLE/PIB and were included in the total population (Table 1). An additional 145 patients were treated off-label with GLE/PIB. The reasons for off-label treatment were a treatment duration shorter or longer than indicated, non-permitted pre-treatment, patients with cirrhosis treated with an off-label duration, hepatic decompensation, and the receipt of other off-label treatment.

The assessment of fibrosis/cirrhosis status was most frequently carried out using a combination of sonography plus laboratory tests to calculate FIB-4/APRI scores (in 46.9% of patients). In 31.4% of patients, an additional elastography assessment was also performed. In 4.7% of patients, elastography and FIB-4/APRI were used. In 15% of patients, the fibrosis assessment was based on FIB-4/APRI and clinical evidence exclusively. Biopsies were performed in 0.8% of patients. The FIB-4 and APRI data were available for all patients.

For the 2354 on-label patients included, the median (range) age was 46 (18–87) years, 1634 patients (69.4%) were male, and 2133 (90.6%) were TN prior to enrollment (Table 1). The median (IQR) length of infection was shorter for patients with active drug use (10.5 (4.5–20)) and HIV coinfection (3 (1–15)) compared with the total population (15 (6–22)) and longer in patients who are treatment-experienced (TE) (20 (11–25)) (Supplementary Figure S1). The baseline comedications are shown in Supplementary Table S1. Overall, 1733 patients (73.6%) had at least one comorbidity, 976 patients (41.5%) had one or more key comorbidities (OST, active drug use, psychiatric disorders, alcohol abuse/dependence, HIV coinfection), and 977 (41.5%) were of migrant status. There was a large degree of overlap between the special populations of interest, with 772 patients (32.8%) falling into two or more of the categories analyzed (Supplementary Table S2). The most common combinations of subgroups were migrants who also received OST (*n* = 191, 8.1%) and those receiving OST who also had a psychiatric comorbidity (*n* = 122, 5.2%). Patient demographics and clinical characteristics were balanced across groups, except for gender, where there was a lower proportion of male patients in the ‘no key comorbidities’ group (63.0%) than in the overall population (69.4%) (Table 1). 

### 3.2. Effectiveness

**Figure 1:**
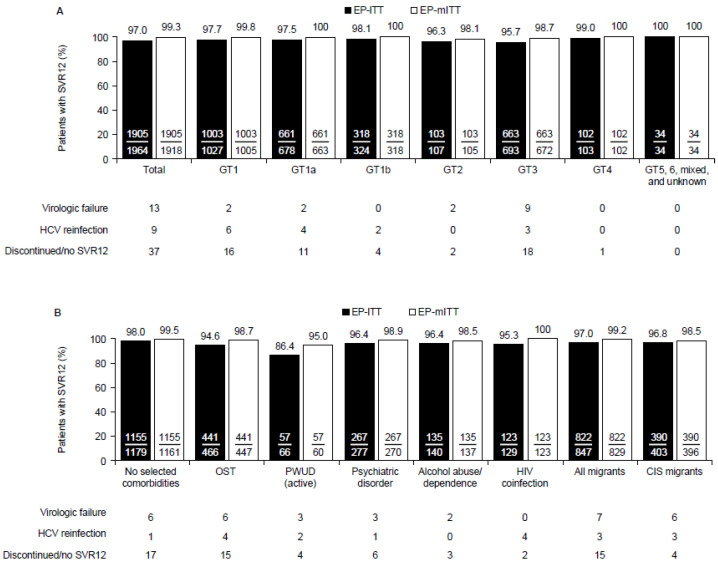
SVR12 rates in the effectiveness population according to (**A**) HCV genotype and (**B**) key comorbidities and migrant status (primary effectiveness endpoint). Patients who discontinued GLE/PIB prematurely and achieved SVR12 were counted as virologic responders (*n* = 11). EP-mITT analysis excluded patients who discontinued GLE/PIB prematurely and did not achieve SVR12, and patients with HCV reinfection. *n* = 258 patients in the EP-ITT population were cirrhotic; 252 (97.7%) of these patients achieved SVR12. CIS, Commonwealth of Independent States (Armenia, Azerbaijan, Belarus, Kazakhstan, Kyrgyzstan, Moldova, Russia, Tajikistan, and Uzbekistan); EP, effectiveness population; GT, genotype; HCV, hepatitis C virus; HIV, human immunodeficiency virus; ITT, intention to treat; mITT, modified intention to treat; OST, opioid substitution therapy; PWUD (active), people who actively use drugs; SVR12, sustained virologic response at PTW12.

Of the 2354 patients, 1964 patients had valid SVR12 data (EP; Supplementary Figure S2). Of these, 1905 (97.0%) achieved SVR12 (primary effectiveness endpoint; Figure 1) according to the EP-ITT analysis, with similar rates across all GTs (95.7% to 100%) (Figure 1a). Of the 59 patients (3.0%) who did not achieve SVR12, 13 (0.7%) had virologic failure, 9 (0.5%) had HCV reinfection, and 37 (1.9%) discontinued and did not achieve SVR12. Of note, 11 patients discontinued therapy prematurely, but did achieve SVR12. The overall SVR12 rate in the EP-mITT analysis was 99.3% (*n*/*N* = 1905/1918). In the EP-ITT analysis, the SVR12 rates were similar across the populations of special interest (94.6% to 97%) excluding PWUD (active) who had an SVR12 rate of 86.4% (*n*/*N* = 57/66). The SVR12 rates in the EP-mITT analysis were similar for all populations of special interest (≥ 95.0%) (Figure 1b). 

**Figure 2:**
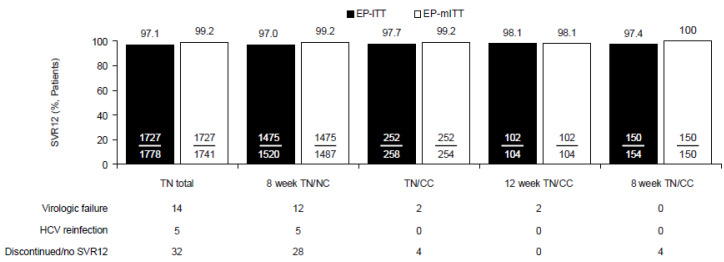
SVR12 rates among TN patients who received GLE/PIB according to cirrhosis status and treatment duration. CC, compensated cirrhosis; EP, effectiveness population; HCV, hepatitis C virus; ITT, intention to treat; mITT, modified intention to treat; TN, treatment-naïve; SVR12, sustained virologic response at PTW12.

In TN patients, the overall EP-ITT SVR12 rate was 97.1% (*n*/*N* = 1727/1778) and remained high regardless of cirrhosis status (TN/NC: *n*/*N* = 1475/1520, 97.0%; TN/CC: *n*/*N* = 252/258, 97.7%) or the duration of therapy (12 weeks, or 8 weeks after the label change). In the TN/CC patients, the EP-ITT SVR12 rates were high with both 12-week (98.1%, n/N = 102/104) and 8-week (97.4%, *n*/*N* = 150/154) GLE/PIB (Figure 2). The SVR12 rates remained >94.6% regardless of GT (Supplementary Figure S3). Similar results were seen in the EP-mITT analysis (Figure 2). 

**Figure 3:**
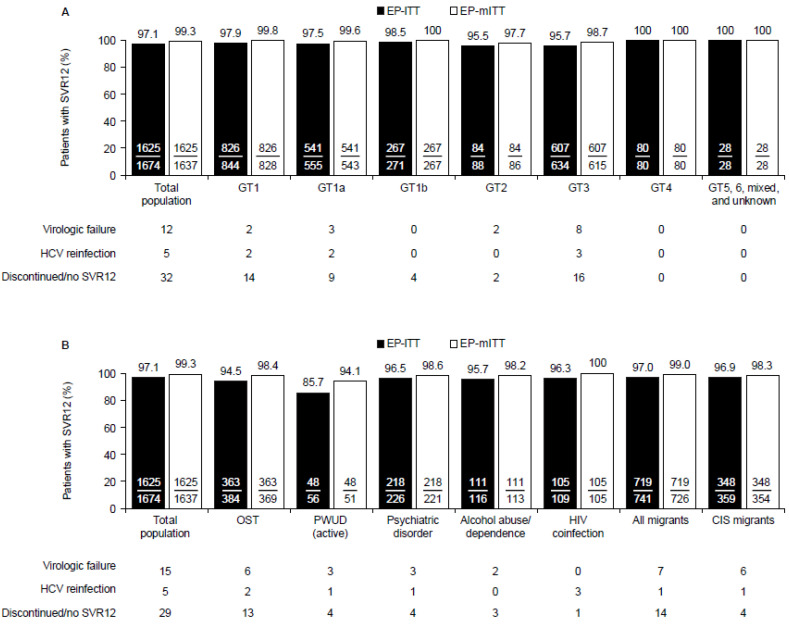
SVR12 rates among TN patients who received GLE/PIB for 8 weeks according to (**A**) HCV genotype and (**B**) key comorbidities and migrant status. Patients who discontinued GLE/PIB prematurely and achieved SVR12 were counted as virologic responders (*n* = 11). EP-mITT analysis excluded patients who discontinued GLE/PIB prematurely and did not achieve SVR12, or patients with HCV reinfection. CIS, Commonwealth of Independent States (Armenia, Azerbaijan, Belarus, Kazakhstan, Kyrgyzstan, Moldova, Russia, Tajikistan, and Uzbekistan); EP, effectiveness population; GT, genotype; HCV, hepatitis C virus; HIV, human immunodeficiency virus; ITT, intention to treat; mITT, modified intention to treat; OST, opioid substitution therapy; PWUD (active), people who actively use drugs; SVR12, sustained virologic response at PTW12.

In TN patients who received 8-week GLE/PIB, the overall EP-ITT SVR12 rate was 97.1% (*n*/*N* = 1624/1674) and the EP-mITT rate was 99.3% (*n*/*N* = 1625/1637) (Figure 3). Compared with the primary effectiveness population, similar patterns of responses were observed across the GTs and populations of special interest. 

In the off-label population, the EP-ITT SVR12 rate was 92.5% (*n*/*N* = 111/120); three patients experienced on-treatment virologic failure, four patients discontinued, and two experienced reinfection. Therefore, the EP-mITT SVR12 rate was 97.4% (*n*/*N* = 111/114). 

### 3.3. Patient-Reported Outcomes

**Figure 4:**
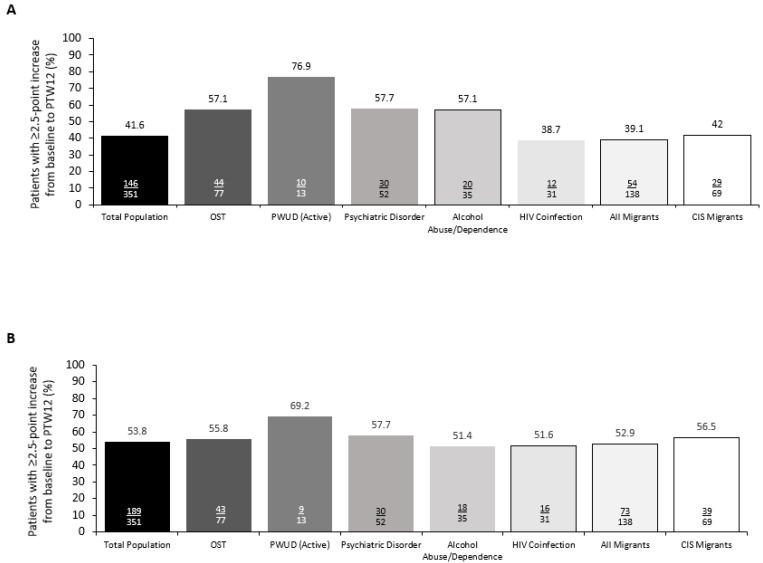
Percentage of patients with a clinically relevant improvement (≥2.5-point increase from baseline to PTW12) in (**A**) SF-36 physical component summary score and (**B**) SF-36 mental component summary score, according to key comorbidities and migrant status. CIS, Commonwealth of Independent States (Armenia, Azerbaijan, Belarus, Kazakhstan, Kyrgyzstan, Moldova, Russia, Tajikistan, and Uzbekistan); HIV, human immunodeficiency virus; OST, opioid substitution therapy; PTW, post-treatment week; PWUD (active), people who actively use drugs.

The PROs (SF-36) were measured at baseline in 889 patients (Table 1 and Figure 4). At baseline, compared with no key comorbidities, the SF-36 PCS scores were lower in patients with OST and the MCS scores were lower in patients with OST, active drug use, psychiatric disorders, and alcohol abuse/dependence (Table 1). Of patients with data available at PTW12, 146/351 patients (41.6%) had a clinically meaningful improvement in PCS score, and 189/351 (53.8%) had a clinically meaningful improvement in MCS score (Figure 4). Of the populations analyzed, patients with active drug use and those with psychiatric disorders had the highest proportion of patients with clinically meaningful improvements (PCS: 76.9% and 57.7%, respectively; MCS: 69.2% and 57.7%, respectively; Figure 4). The mean PCS score continued to increase from baseline to PTW12 for all patients regardless of the comorbidity of interest or migrant status (Supplementary Figure S4a). The mean MCS scores increased from baseline to EOT in groups with all comorbidities of interest and migrant status; however, for patients with active drug use and alcohol abuse, the mean scores decreased from EOT (drug use: 43; alcohol abuse: 42) to PTW12 (drug use: 42; alcohol abuse: 41; Supplementary Figure S4b).

### 3.4. Adherence

At EOT, physicians judged 89.4% of patients to have been 100% compliant with their therapy; Commonwealth of Independent States (CIS (Armenia, Azerbaijan, Belarus, Kazakhstan, Kyrgyzstan, Moldova, Russia, Tajikistan, and Uzbekistan)) migrants were perceived to have the highest rates of total compliance (91.7%) and PWUD (active) were perceived to have the lowest (84.1%; Supplementary Figure S5). 

### 3.5. Safety

AEs were reported in 631/2354 patients (26.8%), with the most commonly reported AEs being fatigue (9.1%), headache (6.0%), and nausea (3.1%). Serious AEs occurred in 44 patients (1.9%) and AEs leading to discontinuation occurred in three patients (0.1%). The safety in the off-label cohort was similar to the on-label cohort (33.1%, *n*/*N* = 48/145), with the most frequent AEs being fatigue (12.4%), headache (5.5%), and pruritus (4.1%) in 145 patients (Table 2). Laboratory abnormalities ≥ 3 × ULN occurred in 0.8% patients (aspartate aminotransferase, alanine aminotransferase, and total bilirubin). No unexpected safety signals were observed (Table 2).

## 4. Discussion

Real-world studies provide valuable information on the effectiveness and safety of treatments in heterogeneous groups that include patients from marginalized populations. The data from this analysis using the DHC-R demonstrate that GLE/PIB was highly effective and well tolerated in routine clinical practice, even in underserved patients. The overall EP-ITT SVR12 rate in this analysis was 97.1%, and after excluding non-virologic failures and HCV reinfections, the overall EP-mITT SVR12 rate was 99.3%. These high rates of SVR were seen regardless of GT and support GLE/PIB data from numerous clinical trials and real-world studies [11,12,21,25,26]. The SVR12 rates in the off-label population were similar to the on-label population. 

High rates of SVR12 were also observed in TN patients with or without compensated cirrhosis (EP-ITT: ≥ 97%); these easier-to-treat patients formed the majority of this study population (90.6%) and an increasing proportion of patients within the overall registry [27]. Importantly, these real-world data showed the comparable effectiveness of GLE/PIB when administered for 8 or 12 weeks in TN/CC patients (ITT: 97.4% and 98.1%, respectively). These SVR rates are consistent with the results from the EXPEDITION-8 trial (97.7% in the ITT population), which led to the addition of the 8-week treatment duration for TN/CC patients in the GLE/PIB label [9,12]. These data also support recent updates in HCV treatment guidelines from AASLD and EASL and provide additional real-world evidence that all TN patients can be effectively treated with 8-week GLE/PIB, regardless of the presence of cirrhosis [5,6]. 

The analysis of the populations of interest conducted in this study showed EP-mITT SVR12 rates of ≥ 95% regardless of comorbidities or migration status. In the EP-ITT analysis, patients with active drug use had a slightly lower SVR12 rate (86.4%): 4/66 patients discontinued prematurely, 3/66 experienced virologic failure, and 2/66 experienced HCV reinfection. These patients also had the lowest adherence of all populations of interest. Previous studies have shown that injection drug use is associated with a loss to follow-up that exceeds virologic failure with DAA therapy [28,29]. Any SVR12 achieved by patients lost to follow-up are unconfirmed and unreported. There are several groups of patients considered difficult to treat and engage with healthcare, including people who inject drugs and those with psychiatric disorders, on OST, with alcohol abuse/misuse disorders, and migrants [30]. To achieve the WHO global HCV elimination goal, engagement with these patient populations is essential [3]. Many of them are also at the highest risk of HCV infection; for example, injection drug use accounts for transmission in over 44% of cases in Europe, and migrants account for approximately 14% of patients with HCV infection in Europe [1,31,32]. This analysis demonstrates the real-world effectiveness of GLE/PIB in all of these marginalized populations, reinforcing guideline recommendations to treat all patients [4,5,6].

Epidemiological data suggest that the characteristics of HCV-infected individuals have changed over the last decade from TE/CC patients to TN/NC patients, which is reflected in the population included in this analysis [33,34]. The duration of HCV infection in the overall patient population is also similar to recently published data, showing that the duration of HCV infection has only slightly reduced since 2014 [27]. Although global data suggest that the number of people who inject drugs living with HCV infection has increased in recent years [32], the number of PWUD (active) included in this analysis was low compared with other comorbidities. This under-representation may reflect the low number of addiction centers included in this study, but could also point to gaps in the care cascade, potentially centered around reaching these patients and engaging them in treatment. Taken together, this emphasizes the need to ensure that HCV treatment is simple, targeted, and patient-centered. The availability of all-oral DAAs, with short treatment durations, has the potential to increase access to care, particularly in underserved populations [21]. In fact, recent studies have shown that 8 weeks of treatment with GLE/PIB demonstrated high efficacy in treating both people who inject drugs [35] and people who have been imprisoned [36].

In addition to the high rates of SVR12, there were also clinically meaningful improvements from baseline in the physical and mental SF-36 component summary scores. At baseline, patients with OST, active drug use, psychiatric disorders, and alcohol abuse/dependence had lower MCS scores than patients with no key comorbidities. This is to be expected due to the high physical and mental burdens associated with these comorbidities. At PTW12, all groups saw clinically meaningful improvements in functioning and wellbeing, with PWUD (active) and patients with psychiatric disorders deriving the greatest benefit. These findings reflect previous studies that have shown that patients with higher mental and social burdens have the greatest improvements in PROs following SVR12 [37,38]. Although improvements were seen in all groups in MCS scores from baseline to PTW12, the mean scores decreased from EOT to PTW12 in patients with active drug use and alcohol abuse. This may be because of the decreased interaction between patients and HCPs once treatment is complete. The improvements in PROs that come with successful virologic response reinforce the importance of treatment beyond clinical cure and highlight that HCV cure can be a catalyst for change in other areas for many patients [8].

This analysis included patients treated with GLE/PIB in clinical practice. However, there are some limitations inherent to real-world studies. Due to the non-interventional nature of this study, post-treatment (PTW12/24) data were not documented for all patients in the safety population, so effectiveness could only be analyzed for patients who had documented data at PTW12/24 (EP). Due to the data analysis split by on-label vs off-label use, it is difficult to directly compare with studies analyzing GLE/PIB efficacy as a function of its duration. Data may have been inconsistently collected or recorded and there may have been recall bias, particularly in the reporting of adherence, PROs, and AEs. AEs are often underreported in real-world settings compared with clinical trials. Some subgroups in this analysis were not well represented and included small numbers of patients (e.g., patients infected with GT5 or GT6). The use of PPIs is likely underestimated in the DHC-R, as only prescribed PPIs are captured. METAVIR Fibrosis stage (F0–4) was often not defined in this patient population because the FibroScan elastography necessary for scoring had to be paid for privately and is not necessary for treatment initiation, and biopsy was performed only rarely in individual cases. 

The findings of this real-world analysis add to the growing body of evidence demonstrating that GLE/PIB is a highly effective and well-tolerated treatment option for a wide range of patient subgroups who will be key for HCV elimination. The results support clinical trial data and highlight the potential of GLE/PIB to contribute to HCV elimination targets, particularly in the treatment of patients deemed to be high-risk who are often unwilling or unable to access care, and for whom the benefits of treatment often extend beyond ‘clinical cure’.

## Figures and Tables

**Table 1:** Demographics and clinical characteristics at baseline (total on-label population).

	Total Population *N* = 2354	Key Comorbidities ^1^						Migrant Status	
		None ^2^ *N* = 1378	OST *N* = 609	PWUD (Active) *N* = 87	Psychiatric Disorder *N* = 322	Alcohol Abuse/ Dependence *N* = 170	HIV Coinfection *N* = 142	All Migrants ^3^ *N* = 977	CIS Migrants *N* = 474
**Male, *n* (%)**	1634 (69.4%)	868 (63.0%)	492 (80.8%)	72 (82.8%)	225 (69.9%)	132 (77.6%)	125 (88.0%)	728 (74.5%)	349 (73.6%)
**Age** **Years, median (range)** **> 65 years**, ***n* (%)**	46 (18–87) 156 (6.6%)	47 (18–87) 131 (11.1)	44 (21–69) 3 (0.5%)	44 (23–65) 0	47 (18–83) 11 (3.4%)	47 (18–66) 1 (0.6%)	44 (37–66) 1 (0.7%)	41 (18–82) 48 (4.9%)	40 (18–82) 17 (3.6%)
**Race, *n* (%)**									
**White** **Other ^4^**	2242 (95.2) 112 (4.8)	1291 (93.7) 87 (6.3)	601 (98.7) 8 (1.3)	86 (98.9) 1 (1.1)	313 (97.2) 9 (2.8)	166 (97.6) 4 (2.4)	132 (93.0) 10 (7.0)	867 (88.7) 110 (11.3)	469 (98.9) 5 (1.1)
**Body weight (kg), median (range)**	75.0 (35.0–180.0)	75.0 (39.3–180.0)	76.0 (40.0–180.0)	72.0 (35.0–143.0)	75.0 (45.5–180.0)	75.0 (45.0–133.0)	71.5 (43.0–121.5)	75.0 (43.0–178.0)	77.4 (43.0–135.0)
**BMI (kg/m^2^), mean (SD)**	25.6 (5.0)	25.8 (4.9)	25.3 (5.6)	24.5 (5.4)	25.6 (5.3)	25.2 (4.6)	23.7 (4.2)	25.9 (4.8)	26.2 (4.6)
**HCV genotype, *n* (%)**									
**1** **1a** **^5^** **1b** **^5^** **other** **^5,6^** **2** **3** **4** **5** **6** **Mixed** **^7^** **Unknown**	1231 (52.3) 841 (68.3) 361 (29.3) 29 (2.4) 131 (5.6) 837 (35.6) 112 (4.8) 2 (0.1) 8 (0.3) 20 (0.8) 13 (0.6)	736 (53.4) 440 (59.8) 277 (37.6) 19 (2.6) 78 (5.7) 474 (34.4) 58 (4.2) 2 (0.1) 7 (0.5) 15 (1.1) 8 (0.6)	293 (48.1) 248 (84.6) 38 (13.0) 7 (2.4) 31 (5.1) 261 (42.9) 18 (3.0) 0 1 (0.2) 2 (0.3) 3 (0.5)	43 (49.4) 39 (90.7) 4 (9.3) 0 6 (6.9) 36 (41.4) 2 (2.3) 0 0 0 0	175 (54.3) 137 (78.3) 35 (20.0) 3 (1.7) 22 (6.8) 105 (32.6) 18 (5.6) 0 0 1 (0.3) 1 (0.3)	85 (50.0) 68 (80.0) 15 (17.6) 2 (2.4) 9 (5.3) 65 (38.2) 7 (4.1) 0 0 3 (1.8) 1 (0.6)	80 (56.3) 69 (86.3) 11 (13.8) 0 6 (4.2) 27 (19.0) 28 (19.7) 0 0 1 (0.7) 0	438 (44.8) 231 (52.7) 193 (44.1) 14 (3.2) 53 (5.4) 407 (41.7) 51 (5.2) 1 (0.1) 8 (0.8) 14 (1.4) 5 (0.5)	193 (40.7) 69 (35.8) 117 (60.6) 7 (3.6) 28 (5.9) 240 (50.6) 2 (0.4) 0 0 7 (1.5) 4 (0.8)
**HCV RNA, median (IQR), Log_10_ IU/mL**									
	6.1 (5.5–6.6)	6.1 (5.5–6.6)	6.1 (5.5–6.7)	6.3 (5.6–6.8)	6.2 (5.4–6.7)	6.1 (5.6–6.7)	6.1 (5.5–6.7)	6.1 (5.5–6.6)	6.0 (5.4–6.6)
**Platelets per μL, median (range) ^8^**	218,000 (17,500–616,000)	220,000 (31,000–616,000)	210,000 (17,500–564,000)	198,000 (47,000–362,000)	224,500 (79,000–564,000)	207,000 (51,000–468,000)	210,000 (17,500–372,000)	219,000 (45,000–557,000)	223,000 (51,000–369,000)
**Non-cirrhotic ^9^**	2033 (86.4%)	1211 (87.9%)	507 (83.3%)	74 (85.1%)	279 (86.6%)	126 (74.1%)	119 (83.8%)	870 (89.0%)	432 (91.1%)
**METAVIR Fibrosis stage, *n* (%)**									
**F0–F1** **F2** **F3** **F4** **Missing stage data**	469 (19.9) 191 (8.1) 58 (2.5) 321 (13.6) 1315	264 (19.2) 116 (8.4) 34 (2.5) 167 (12.1) 797	116 (19.0) 47 (7.7) 13 (2.1) 102 (16.7) 331	17 (19.5) 8 (9.2) 3 (3.4) 13 (14.9) 46	73 (22.7) 23 (7.1) 12 (3.7) 43 (13.4) 171	38 (22.4) 19 (11.2) 7 (4.1) 44 (25.9) 62	36 (25.4) 8 (5.6) 3 (2.1) 23 (16.2) 72	182 (18.6) 67 (6.9) 26 (2.7) 107 (11.0) 595	72 (15.2) 34 (7.2) 15 (3.2) 42 (8.9) 311
**HCV TN**	2133 (90.6%) ^9^	1257 (91.2%)	551 (90.5%)	81 (93.1%)	284 (88.2%)	159 (93.5%)	125 (88.0%)	900 (92.1%)	443 (93.5%)
**Estimated duration of infection (years), mean (SD)**									
	15.7 (11.3)	17.1 (12.5)	14.2 (8.7)	12.3 (8.9)	14.9 (10.4)	15.5 (9.6)	8.0 (9.9)	13.5 (10.1)	14.3 (8.9)
**Employment status, *n* (%)**									
**Employed** **Unemployed** **Unknown**	952 (40.4) 1015 (43.1) 387 (16.4)	658 (47.8) 500 (36.3) 220 (16.0)	143 (23.5) 358 (58.8) 108 (17.7)	20 (23.0) 50 (57.5) 17 (19.5)	81 (25.2) 191 (59.3) 50 (15.5)	53 (31.2) 85 (50.0) 32 (18.8)	68 (47.9) 46 (32.4) 28 (19.7)	476 (48.7) 363 (37.2) 138 (14.1)	244 (51.5) 169 (35.7) 61 (12.9)
**Suspected route of transmission, *n* (%)**									
**Blood products** **Drugs (IV, nasal)** **Sexual transmission** **Surgical/medical procedure** **Other** **Unknown**	169 (7.2) 1178 (50.0) 138 (5.9) 103 (4.4) 64 (2.7) 702 (29.8)	145 (10.5) 432 (31.3) 54 (3.9) 94 (6.8) 52 (3.8) 601(43.6)	5 (0.8) 568 (93.3) 12 (2.0) 1 (0.2) 4 (0.7) 19 (3.1)	1 (1.1) 77 (88.5) 1 (1.1) 1 (1.1) 1 (1.1) 6 (6.9)	15 (4.7) 212 (65.8) 28 (8.7) 6 (1.9) 7 (2.2) 54 (16.8	4 (2.4) 128 (75.3) 8 (4.7) 1 (0.6) 3 (1.8) 26 (15.3)	2 (1.4) 58 (40.8) 62 (43.7) 0 0 20 (14.1)	70 (7.2) 376 (38.5) 42 (4.3) 70 (7.2) 36 (3.7) 383 (39.2)	29 (6.1) 205 (43.2) 14 (3.0) 41 (8.6) 16 (3.4) 169 (35.7)
**Mean SF-36 mental component summary score ^10^**									
	39	42	34 ^11^	33 ^11^	31 ^12^	37 ^12^	41	40	40
**Mean SF-36 physical component summary score ^10^**									
	49	50	48 ^11^	48	48	48	49	50	50

^1^ Patients may have more than one key comorbidity, meaning values do not add up to 2354. ^2^ No key comorbidities defined as no OST, no active drug use, no psychiatric disorders, no alcohol abuse/dependence, no HIV coinfection. ^3^ Country of origin (> 2% migrants) reported as Russia, *n* = 321; Poland *n* = 80; Kazakhstan, *n* = 55; Turkey, *n* = 54; Italy, *n* = 46; Pakistan, *n* = 26; Ukraine, *n* = 23; Romania, *n* = 23. ^4^ Other includes: Black, Asian, and Other. ^5^ Percentages calculated from the number of patients with HCV GT1 in each phase. ^6^ Other GT1 subtype, mixed GT1 subtype or not specified. ^7^ Mixed genotypes (GT1 + GT2, GT1 + GT3, GT1 + GT4, or GT3 + GT4). ^8^ Data available for: total population, *n* = 2209; no key comorbidities, *n* = 1269; HCV TN non-cirrhotic, *n* = 1321; OST, *n* = 423; active drug use, *n* = 47; psychiatric disorders, *n* = 235; alcohol abuse/dependence, *n* = 99; HIV co-infection, *n* = 102. ^9^ *n* = 1837 patients (78.0%) were treatment-naïve and non-cirrhotic and went on to receive GLE/PIB for 8 weeks; *n* = 592 patients (25.1%) were TN/CC, of these *n* = 120 (5.1%) went on to receive GLE/PIB for 12 weeks, and *n* = 176 (7.5%) for 8 weeks. Duration of treatment data were unavailable for 296 TN/CC patients (12.6%). ^10^ Data available for: total population, *n* = 889; no key comorbidities, n = 513; OST, *n* = 207; active drug use, *n* = 34; psychiatric disorders, *n* = 152; alcohol abuse/dependence, *n* = 79; HIV coinfection, *n* = 66; all migrants, *n* = 343; CIS migrants, *n* = 153. ^11^ *p* < 0.05 vs no key comorbidities group. ^12^ *p* < 0.001 vs no key comorbidities group. CIS, Commonwealth of Independent States (Armenia, Azerbaijan, Belarus, Kazakhstan, Kyrgyzstan, Moldova, Russia, Tajikistan, and Uzbekistan); GT, genotype; HCV, hepatitis C virus; HIV, human immunodeficiency virus; IQR, interquartile range; IV, intravenous; OST, opioid substitution therapy; PWUD (active), people who actively use drugs; RNA, ribonucleic acid; SF-36, 36-Item Short Form Health Survey; TN, treatment-naïve.

**Table 2:** Treatment-emergent adverse events and post-baseline laboratory abnormalities (safety population).

	Patients (*N* = 2354)
Adverse events	
Any AE	631 (26.8)
Any serious AE ^1^	44 (1.9)
Any AE leading to study drug discontinuation ^2^	3 (0.1)
**AEs occurring in ≥ 1% of patients**	
Fatigue	215 (9.1)
Headache	142 (6.0)
Nausea	74 (3.1)
Abdominal discomfort	51 (2.2)
Pruritus	45 (1.9)
Diarrhea	38 (1.6)
Arthralgia	27 (1.1)
Deaths	4 (0.2)
**Laboratory abnormalities**	
Alanine aminotransferase, any grade	2144 (91.1)
>5 × ULN	3 (0.1)
Aspartate aminotransferase, any grade	2007 (85.3)
>5 × ULN	5 (0.2)
Total bilirubin, any grade	1918 (81.5)
>5 × ULN	7 (0.4)

Data are *n* (%). ^1^ Serious AEs according to MedDRA-preferred terms were limb abscess, anemia, atrial flutter, B-cell small lymphocytic lymphoma, cardiac failure, cerebrovascular accident, circulatory collapse, colitis, coronary artery disease, dependence, detoxification, drug dependence, drug withdrawal syndrome, dyspnea, endoscopic retrograde cholangiopancreatography, gastroenteritis, headache, hepatectomy, hepatic cirrhosis, hospitalization, humerus fracture, injection-site abscess, intracranial aneurysm, knee operation, Ménière’s disease, multi-organ failure, myocardial infarction, nausea, osteoporosis, pancreatic carcinoma, pleural effusion, renal colic, suicide attempt, thoracic vertebral fracture, toxicity to various agents, vestibular disorder, all *n* = 1; vomiting, *n* = 2, pneumonia, *n* = 2; hepatic neoplasm, *n* = 4. ^2^ AEs leading to study drug discontinuation were due to nausea, *n* = 1; diarrhea, *n* = 1; and vomiting, *n* = 1. AE, adverse event; ULN, upper limit of the normal range.

## Data Availability

Access to individual datasets is not available due to the informed consent. Upon request, aggregated data can be made available.

## Supplementary Materials

The following supporting information can be downloaded at: https://www.mdpi.com/article/10.3390/v14071541/s1, Table S1: Comedication during HCV therapy in >3% patients (ATC Level 4, chemical subgroup), Table S2: Overlap between OST or active drug use, and other populations of special interest (total population), Figure S1: Estimated length of HCV infection, Figure S2: Patient disposition, Figure S3: SVR12 rates in treatment-naïve patients with CC by genotype for (A) 8-week treatment with G/P and (B) 12-week treatment with G/P, Figure S4: Mean SF-36 scores from baseline to PTW12 (A) Physical component summary score (B) Mental component summary score. Figure S5: Compliance of patients who completed treatment, as judged by physicians at the end of therapy.
