# Supplementary material for: Real-World Safety, Effectiveness, and Patient-Reported Outcomes in Patients with Chronic Hepatitis C Virus Infection Treated with Glecaprevir/Pibrentasvir: Updated Data from the German Hepatitis C-Registry (DHC-R)
Source: Viruses. 2022 Jul 14;14(7):1541. doi: 10.3390/v14071541 (PMC9318383; doi:10.3390/v14071541)
Supplement: Supplementary file 1 [file viruses-14-01541-s001.zip › viruses-1733380-supplementary.pdf]

Table S1. Comedication during HCV therapy in >3% patients (ATC Level 4, chemical subgroup).

	**ITT total %**
N07BC: Drugs used in opioid dependence	25.9%
C07AB: Beta blocking agents, selective	6.1%
N02AE: Oripavine derivatives	5.7%
C09AA: ACE inhibitors, plain	5.7%
A02BC: Proton pump inhibitors	5.6%
H03AA: Thyroid hormones	4.4%
C08CA: Dihydropyridine derivatives	4.0%
M02AA: Anti-inflammatory preparations, non-steroids for topical use	3.9%
M01AE: Propionic acid derivatives	3.7%
A11CC: Vitamin D and analogues	3.7%
B01AC: Platelet aggregation inhibitors excl. heparin	3.6%
G02CC: Anti-inflammatory products for vaginal administration	3.5%
N02BA: Salicylic acid and derivatives	3.5%
A01AD: Other agents for local oral treatment	3.5%
N06AX: Other antidepressants	3.4%

ATC, Anatomic Therapeutic Chemical; ACE, angiotensin-converting enzyme.

Table S2. Overlap between OST or active drug use, and other populations of special interest (total population).

**Combination of subgroups**	**Number of patients (*N* = 2354)**
OST + alcohol use	71
OST + active drug use	69
OST + psychiatric comorbidity	122
Alcohol use + PWUD (active)	22
Alcohol use + psychiatric comorbidity	46
PWUD (active) + psychiatric comorbidity	27
OST + alcohol use + PWUD (active)	16
OST + alcohol use + psychiatric comorbidity	20
Alcohol use + PWUD (active) + psychiatric comorbidity	6
OST + alcohol use + PWUD (active) + psychiatric comorbidity	4
Migrants + PWUD (active)	26
CIS-migrants + PWUD (active)	12
Migrants + OST	191
CIS-migrants + OST	105
Migrants + PWUD (active) + OST	24
CIS-migrants + PWUD (active)+ OST	11

CIS, Commonwealth of Independent States; OST, opioid substitution therapy; PWUD (active), people who actively use drugs.

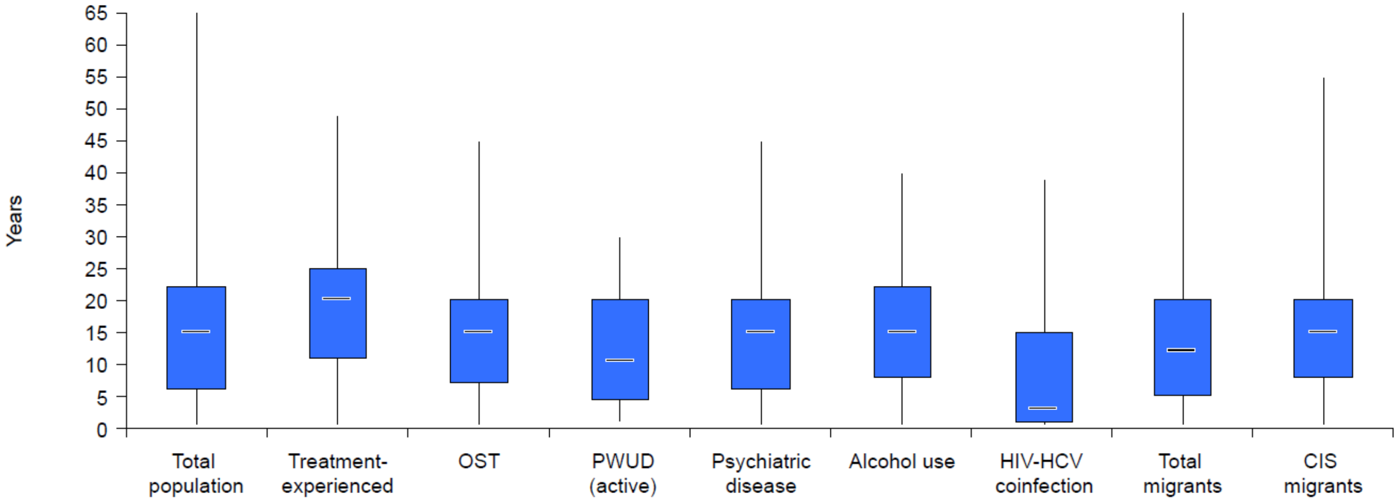

Figure S1. Estimated length of HCV infection.

Data are median (IQR) with min and max values. CIS, Commonwealth of Independent States; HIV, human immunodeficiency virus; OST, opioid substitution therapy; PWUD (active), people who actively use drugs.

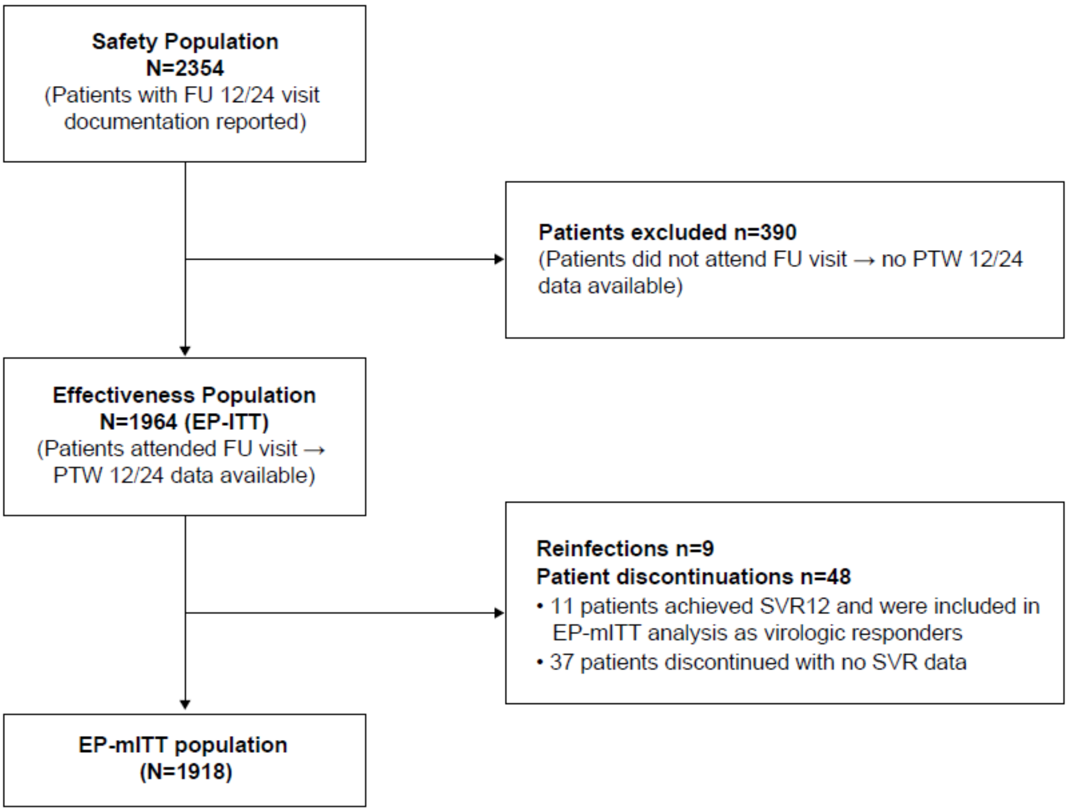

Figure S2. Patient disposition.

EP-ITT, intention-to-treat in the effectiveness population; EP-mITT, modified ITT in the effectiveness population FU, follow-up; PTW, post-treatment week; SVR12, sustained virologic response at post-treatment Week 12

A

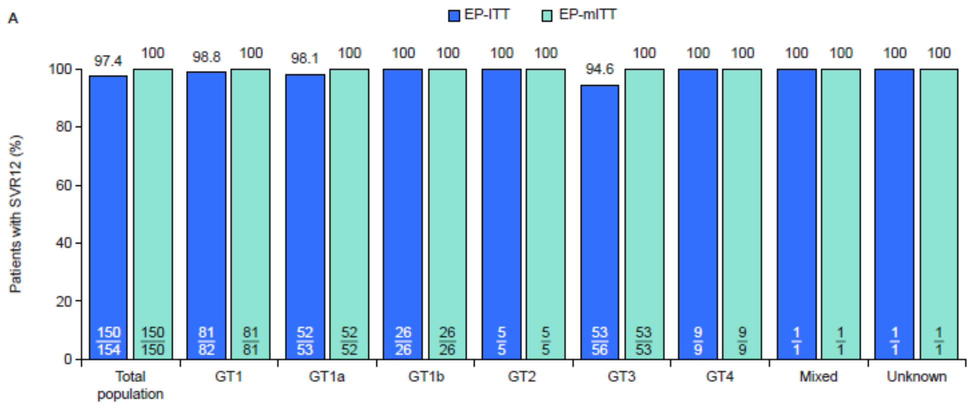

	Total population	GT1	GT1a	GT1b	GT2	GT3	GT4	Mixed	Unknown
Virologic failure	0	0	0	0	0	0	0	0	0
HCV reinfection	0	0	0	0	0	0	0	0	0
Discontinued / no SVR12	4	1	1	0	0	3	0	0	0

B

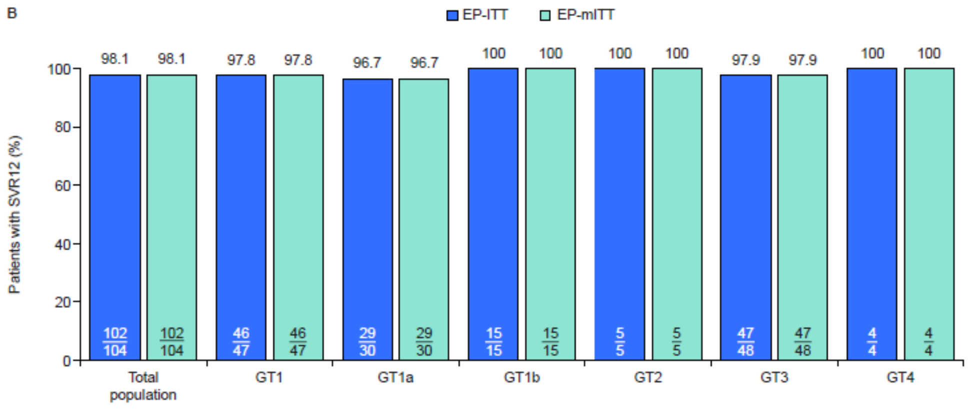

	Total population	GT1	GT1a	GT1b	GT2	GT3	GT4
Virologic failure	2	1	1	0	0	1	0
HCV reinfection	0	0	0	0	0	0	0
Discontinued / no SVR12	0	0	0	0	0	0	0

Figure S3. SVR12 rates in treatment-naïve patients with CC by genotype for (**A**) 8-week treatment with G/P and (**B**) 12-week treatment with G/P.

CC, compensated cirrhosis; G/P, glecaprevir/pibrentasvir; GT, genotype; HCV, hepatitis C virus; ITT, intention to treat; mITT, modified ITT; SVR, sustained virologic response at post-treatment Week 12.

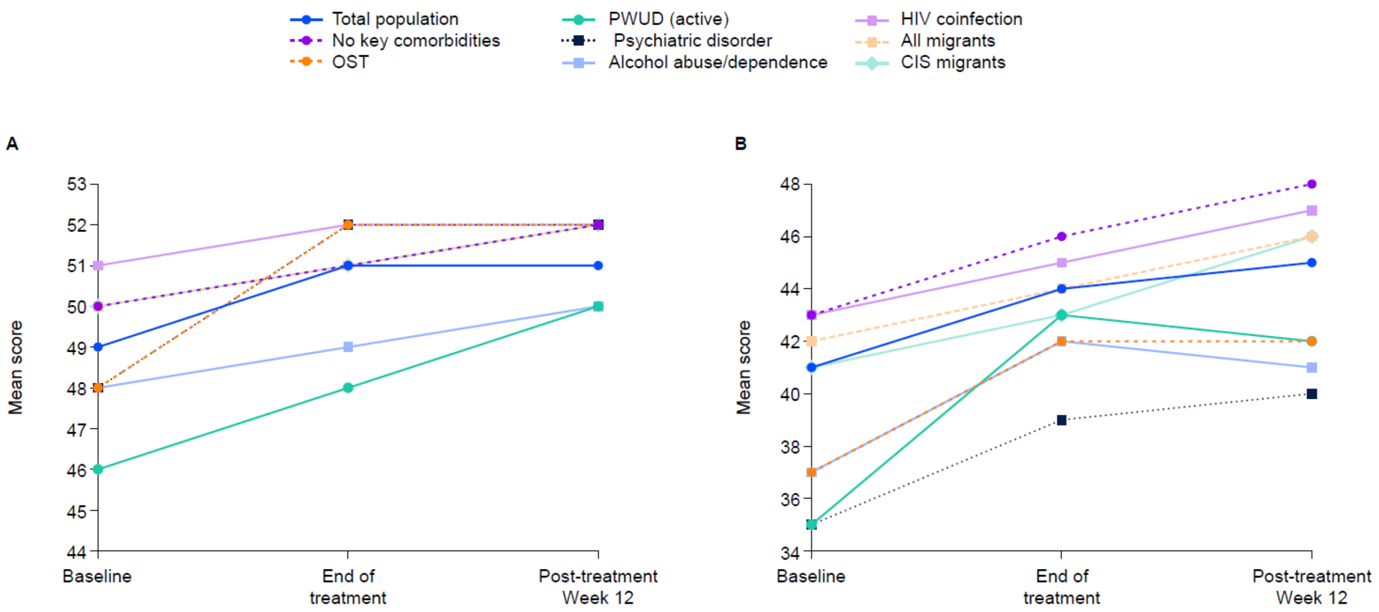

Figure S4. Mean SF-36 scores from baseline to PTW12 (**A**) Physical component summary score (**B**) Mental component summary score.

CIS, Commonwealth of Independent States; HCV, hepatitis C virus; HIV, human immunodeficiency virus; OST, opioid substitution therapy; PWUD (active), people who actively use drugs.

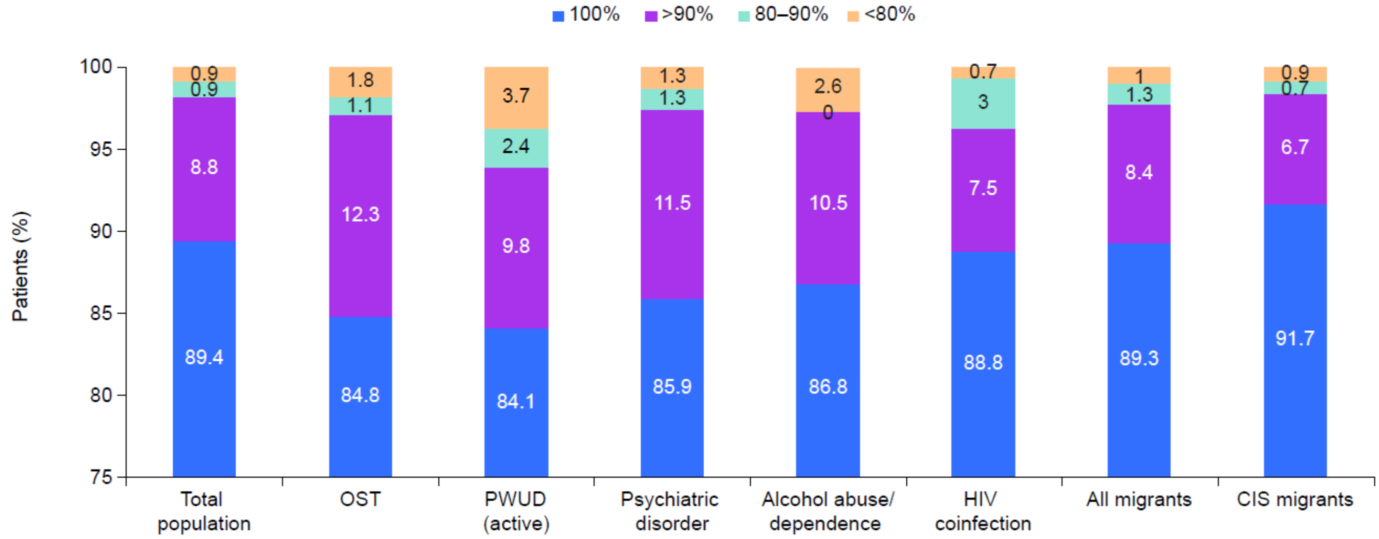

Figure S5. Compliance of patients who completed treatment, as judged by physicians at the end of therapy.

CIS, Commonwealth of Independent States; HIV, human immunodeficiency virus; OST, opioid substitution therapy; PWUD (active), people who actively use drugs.
